# Sense of Coherence among Older Adult Residents of Long-Term Care Facilities in Taiwan: A Cross-Sectional Analysis

**DOI:** 10.1371/journal.pone.0146912

**Published:** 2016-01-11

**Authors:** Ruo-Nan Jueng, Der-Chong Tsai, I-Ju Chen

**Affiliations:** 1 Institute and Department of Nursing, National Yang-Ming University, Taipei, Taiwan; 2 Department of Nursing, National Yang-Ming University Hospital, Yilan, Taiwan; 3 National Yang-Ming University School of Medicine, Taipei, Taiwan; 4 Department of Ophthalmology, National Yang-Ming University Hospital, Yilan, Taiwan; Federal University of Rio de Janeiro, BRAZIL

## Abstract

**Background:**

Growing evidence shows that sense of coherence (SOC) is related to health promotion. Knowledge of SOC among older adults in Taiwan is limited. The present study aimed to investigate SOC status and its relationship to personal and environmental factors among older adult residents of long-term care facilities (LTCFs) in northeastern Taiwan.

**Methods:**

This cross-sectional study was performed in Yilan, Taiwan. With face-to-face interviews, we obtained data from 104 LTCF residents (aged 65 years and older) using the Chinese version of Antonovsky's short 13-item SOC scale. We also collected the information on personal characteristics, physical and social environmental resources. Multiple linear regression was used to analyze factors potentially influencing SOC.

**Results:**

Of the participants, the mean score (±standard deviation) of SOC was 58.3 (±8.8), while scores on SOC subscales (comprehensibility, manageability, and meaningfulness) were 23.4 ±4.5, 17.9 ±3.8, and 17.0 ±3.2, respectively. Education level, activities of daily living and number of LTCF staff were found to be independently associated with SOC status after adjusting for demographic characteristics, health status, and environmental resources. In addition, interactions between personal and environmental factors had a crucial influence on SOC status.

**Conclusions:**

Participants in this study had relatively low SOC scores compared to their counterparts in Western countries. In addition to personal factors, environmental factors can play a significant role in SOC status among older adult LTCF residents. Comprehensive evaluation of SOC status should consider person-environment interaction effects.

## Background

Changes to both family and socioeconomic structures in rapidly aging populations have dramatically increased the demand for long-term care facilities (LTCFs) [1]. It is indicated that health maintenance and promotion of successful aging, in physical, mental, and social terms, among elderly LTCF residents are now top priorities for healthcare providers [2]. Compared to those living in communities, older adult LTCF residents may face additional challenges related to changes in the living environment, daily lifestyle, social networks, and support after moving into an LTCF, potentially resulting in a sense of loss or suffering [3]. Therefore, the importance of enhancing their coping resources has been recognized. These resources may include any characteristic of a person, group, or environment that can facilitate adaptation to stress [4,5]. Sense of coherence (SOC) has been proven to be an influencing factor of coping [6]. Further, Eriksson and Lindström have shown that SOC is an important contributor to the development and maintenance of physical and mental health and quality of life [7,8].

Antonovsky first introduced the concept of SOC in the salutogenic theory as a way of seeing the world that facilitates successful coping with stressors in diverse situations and cultures [4,9]. SOC facilitates an individual’s use of various resources when faced with complex environments and challenges. By influencing the perception of internal and external environmental stimuli, SOC allows an individual to maintain a high degree of health, through a more effective handling of, and a subsequent decrease in, stress [4,10]. SOC includes three core components: comprehensibility, manageability, and meaningfulness. Comprehensibility is the degree to which events are perceived to be explicable, predictable, and structured. Manageability is the degree to which one feels they can cope. Meaningfulness is how much one feels that life makes sense, and how worthy challenges are of investment and engagement [4]. Thus SOC can be understood to represent an individual’s ability to maintain a positive attitude while exhibiting understanding amidst challenging situations (comprehensibility), applying diverse resources (manageability), and seeking and realizing meaning in life (meaningfulness), all of which can help maintain an ideal state of health.

For older adults, SOC has been studied extensively in different settings including amongst community-dwellers, in-hospital patients, and nursing home residents [11–16]. Previous studies on SOC among older adults have focused mainly on its relationship to physiological concerns, psychological status, quality of life, well-being, life satisfaction, and personal and social coping resources. It has been reported that anxiety, fatigue, loneliness, anger, low morale, despair, depression, stress, awareness, low social support, low emotional support, and post-traumatic stress syndrome are all negatively correlated with SOC [13,17,18]. Meanwhile, age, optimism, participation in activities, and perceived health status are positively correlated with SOC [13,17,19,20]. SOC is also a factor in predicting feelings of emotional and social isolation [21], and is able to predict optimism, self-esteem, low depressive mood, self-efficacy, and social support [20,22]. Although a wealth of research has examined SOC as an indicator of psychological health, knowledge regarding factors influencing SOC among older adult LTCF residents is limited, especially in terms of environmental factors and person-environment interactions. It is well known that environmental factors have an important influence on human behavior in general. Further, Lawton’s ecological model of aging emphasizes the potential of a balance between personal competence and environmental demand for producing stress-resistance capabilities and positive feelings of experiences, especially during interactions between older adults and the social environment [23].

Because of changes of life patterns, moving into a LTCF can be a challenge for older adults. SOC may change when people begin new patterns of life experiences [10,16]. Given SOC’s role in health promotion, it is worthy to explore influencing factors for SOC and even developing programs to strengthen SOC. In fact, environmental factors are often more readily modifiable than personal factors. Additionally, there is a knowledge gap regarding Taiwanese older adults’ SOC status. Therefore, we conducted this cross-sectional pilot study to investigate the relationship between SOC status, personal factors, and environmental (physical and social) factors among older adult LTCF residents in northeastern Taiwan. The specific aims of this study were 1) to determine the SOC status among older adult LTCF residents; 2) to determine the factors associated with SOC status; and 3) to examine interaction effects between personal and environmental factors on SOC status. Based on the salutogenic theory and the ecological model of aging, our hypothesis is that environmental factors may play a role in determining SOC status and may modify the relationship between personal factors and SOC status among older adults in LTCFs.

## Methods

### Design, Settings and Participants

This study adopted a cross-sectional research design and employed a face-to-face interview with a structured questionnaire, which was carried out in the LTCFs while participants were in good mental condition. This study was approved by the Institutional Review Board of National Yang-Ming University Hospital (IRB No: 2010045). Data were collected from January 1 to December 31, 2011.

According to the relevant laws and regulations in Taiwan [24], LTCFs’ accommodation capacity is limited to 5 to 200 beds. Those with 49 beds or fewer are recognized as small sized facilities. LTCF must also follow the mandatory staffing ratio regulations. During data collection, there were 39 officially certified LTCFs (10 large sized and 29 small sized) in Yilan County, and a total of 18 (46.2%) facilities were conveniently sampled and visited. Of those, three were large sized facilities with 100 beds or more, and the others were small size facilities with 49 beds or fewer. The inclusion criteria for participants included: over 65 years of age, having resided in the LTCF for more than one month, being communicable, and no history of previously diagnosed mental disorder (e.g. dementia or depression). There were 229 residents met the inclusion criteria when we visited. The authors collected the sample by asking for volunteers from all eligible residents, enrolling 122 eligible and volunteer subjects after obtaining informed consent. The authors documented informed consent by the use of a written consent form and signed by the subject or the subject's legally authorized representative. To increase the likelihood of removing subjects with severe cognitive impairment, we further excluded 18 participants with scores ≤ 17 on the Mini-Mental State Examination (MMSE) from the analysis.

### Instruments

The structured questionnaire was composed of four parts: personal factors, physical environmental factors, social environmental factors, and the SOC scale.

Personal data included demographic data (age, gender, marital status, education, and religion), health status (multi-morbidity, activities of daily living [ADL], MMSE, geriatric depression scale [GDS]), and willingness to be admitted. Data on physical environmental factors covered room type, natural window views, and outdoor public spaces. Social environmental factors included length of LTCF stay (months), number of leisure activities per week, number of family visits per week, and number of LTCF staff (including full-time registered nurses, nurse aides and social workers). Because the distribution of number of staff was bimodal among these 18 sampled LTCFs, number of staff was coded as a binary variable according to the lower limit (31 persons) of large sized facilities.

This study used the Chinese version of Antonovsky's short 13-item SOC scale, the Chinese SOC scale, which was validated by Tang and Dixon (Cronbach’s α = 0.89) [25]. The short-form SOC scale has been proven to be nearly equivalent to the long form in its reliability and validity [26]. The short-form SOC scale is a 7-point Likert scale with 5 items for comprehensibility, 4 for manageability, and 4 for meaningfulness, with each item rated on a scale from 1 (never) to 7 (very often) and a sum score ranging from 13 to 91. Higher scores indicate stronger SOC.

### Data analysis

Statistical analyses were performed with SPSS 20.0 and p<0.05 was set as the required level of significance. All data were expressed as percentage or mean ± standard deviation (SD). Independent t test, one-way ANOVA with Tukey’s post-hoc test and Pearson’s correlation analysis were used to explore the influences of personal factors, physical and social environmental factors on SOC and its three core components. Only the significant influencing variables were entered as covariates into the multiple regression models. To investigate potential associations between personal factors (demographic characteristics and health status), environmental factors (physical and social) and SOC, multiple regression analyses were conducted with overall SOC scores as the dependent variable. To estimate how, and to what extent, environmental factors influenced SOC, a hierarchical regression was performed. Personal and environmental factors were entered block-wise into the models. Model 1 considered personal factors as independent variables, whereas environmental factors were added in Model 2. To examine the interaction effects between personal and environmental factors on SOC, a multiple regression with a stepwise selection method was performed in Model 3 using all significant influencing variables and their interactions. The stepwise method selected a subset of the variables that had a high correlation with SOC and identified the model that explained the greatest amount of variation in SOC. Multicollinearity diagnostics were assessed on the basis of the suggestions in the SPSS linear regression model. The Akaike information criterion (AIC) value of each model was also calculated.

## Results

One hundred and four eligible participants (64 [61.5%] females) were finally recruited into the study. Table 1 shows the distribution of individual characteristics, environmental resources and SOC scores (total scale and three sub-scales). The mean age was 77.3 ±6.9 years (range: 65–93). Generally, males were younger and had higher level of education, and a higher proportion of males were married. The mean ADL, MMSE, and GDS scores of all participants was 62.3 ±5.8 (range: 28–88), 22.6 ±1.5 (range: 21–27), and 8.1 ±1.7 (range: 4–15), respectively. The mean number of leisure activities and family visits per week was 4.3 ±1.0 (range: 3–6) and 2.3 ±0.6 (range: 1–3), respectively. The mean scores of the Chinese SOC scale and its three subscales (comprehensibility, manageability, and meaningfulness) for all participants were 58.3 ±8.8 (range: 34–79), 23.4 ±4.5 (range: 11–34), 17.9 ±3.8 (range: 10–27), and 17.0 ±3.2 (range: 6–24).

**Table 1:** Characteristics and sense of coherence of participants (n = 104).

Variables	
**Personal Factor**	
Age, mean years (SD)	77.3 (6.9)
Gender, n (%)	
Female	64 (61.5)
Male	40 (38.5)
Marital status, n (%)	
Unmarried	10 (9.6)
Married	54 (51.9)
Divorced or widowed	40 (38.5)
Education level, n(%)	
Illiterate	52 (50.0)
Elementary school	37 (35.6)
High school & above	15 (14.4)
Religion, n (%)	
No	14 (13.5)
Yes	90 (86.5)
Multi-morbidity, n (%)	
1 chronic disease	7 (6.7)
2 chronic diseases	39 (37.5)
3 chronic diseases	26 (25.0)
4 chronic diseases and above	32 (30.8)
Activities of daily living, mean (SD)	62.3 (5.8)
Mini-mental status examination, mean (SD)	22.6 (1.5)
Geriatric depression scale, mean (SD)	8.1 (1.7)
Voluntary admission, n(%)	
Yes	24 (23.1)
No	80 (76.9)
**Physical Environmental Factor**	
Room type, n (%)	
Single room	12 (11.5)
Double room	21 (20.2)
≥3 persons/room	71 (68.3)
Natural window views, n (%)	
Yes	76 (73.1)
No	28 (26.9)
Outdoor public space, n (%)	
Yes	35 (33.7)
No	69 (66.3)
**Social Environmental Factor**	
Length of LTCF stay, mean months (SD)	17.2 (17.5)
Number of leisure activities per week, n (%)	
3 per week	23 (22.1)
4 per week	46 (44.2)
5 per week	17 (16.3)
6 per week	18 (17.3)
Number of family visits per week, n (%)	
1 per week	4 (3.8)
2 per week	61 (58.7)
3 per week	39 (37.5)
Number of LTCF staff, n (%)	
<31	76 (73.1)
≥31	28 (26.9)
**Sense of Coherence**, mean (SD)	
Total score	58.3 (8.8)
Comprehensibility subscore	23.4 (4.5)
Manageability subscore	17.9 (3.8)
Meaningfulness subscore	17.0 (3.2)

SD: standard deviation; LTCF: long-term care facility.

Table 2 presents the factors influencing SOC as determined by one-way ANOVA test, independent t test or Pearson’s correlation analysis. The effects of education level on SOC (F = 5.99, p = 0.003), comprehensibility (F = 3.61, p = 0.031) and meaningfulness (F = 7.29, p = 0.001) were significant. Tukey’s post-hoc tests indicated that subjects with a high school and above education level had significantly higher scores in SOC, comprehensibility and meaningfulness than illiterate subjects. ADL was significantly correlated with SOC (r = 0.31, p = 0.001), comprehensibility (r = 0.26, p = 0.008) and manageability (r = 0.35, p = 0.001). Residents in LTCFs with ≥31 staff members had significantly higher scores in SOC (t = -3.36, p = 0.001), manageability (t = -2.54, p = 0.015) and meaningfulness (t = -3.01, p = 0.003) than those in LTCFs with <31 staff members. In addition, age, marital status, multimorbidity, voluntary admission, outdoor public space, and frequency of leisure activities were also significantly associated with SOC or any one of the three core components.

**Table 2:** Factors influencing sense of coherence and three core components.

	Sense of coherence		Comprehensibility		Manageability		Meaningfulness	
	Mean (SD)	*F/t/r*^§^	Mean (SD)	*F/t/r*^§^	Mean (SD)	*F/t/r*^§^	Mean (SD)	*F/t/r*^§^
**Personal Factor**								
Age		0.22*		0.21*		0.20*		0.06
Marital status		4.82*c>b		5.20**c>b		4.08*c>b		1.00
Unmarried (a)	58.5(8.2)		23.1(5.3)		17.2(3.7)		18.2(2.9)	
Married (b)	56.0(7.6)		22.2(4.4)		17.1(3.0)		16.7(2.8)	
Divorced or widowed (c)	61.5(9.5)		25.1(3.9)		19.2(4.5)		17.2(3.6)	
Education level		5.99**c>a		3.61*c>a		1.57		7.29**c>b, a
Illiterate (a)	56.0(8.8)		22.4(4.6)		17.3(3.4)		16.4(3.2)	
Elementary school (b)	59.2(8.3)		24.0(3.8)		18.3(4.3)		16.9(2.9)	
High school & above (c)	64.3(6.9)		25.6(5.0)		19.1(3.5)		19.7(2.3)	
Multi-morbidity		2.27		1.47		0.53		3.25*
1 chronic disease	53.6 (7.6)		21.0(3.9)		17.9(4.5)		14.7(1.6)	
2 chronic diseases	60.3(9.5)		24.2(4.8)		18.4(4.1)		17.7(2.8)	
3 chronic diseases	55.7(8.5)		22.5(4.8)		17.2(3.1)		16.0(3.7)	
4 chronic diseases and above	59.2(7.7)		23.8(3.8)		17.9(3.9)		17.5(2.9)	
Activity of daily living		0.31**		0.26**		0.35**		0.09
Voluntary admission		0.56		-2.24*		0.03		-1.59
Yes	55.7(9.8)		21.7(4.3)		17.9(5.0)		16.1(2.9)	
No	59.1(8.4)		24.0(4.4)		17.9(3.4)		17.3(3.2)	
**Physical Environment Factor**								
Outdoor public space		2.39*		1.59		2.09*		2.04*
Yes	61.5(10.5)		24.5(5.5)		19.1(4.6)		17.9(3.4)	
No	56.7(7.3)		22.9(3.8)		17.3(3.2)		16.6(2.9)	
**Social Environment Factor**								
Number of leisure activities per week		5.02**c, d, a>b		4.70**c, a>b		2.65		2.06
3 per week (a)	60.6(7.2)		25.2(2.6)		18.1(3.9)		17.3(2.7)	
4 per week (b)	54.8(6.7)		21.7(3.8)		16.9(2.7)		16.2(3.0)	
5 per week (c)	62.1(10.3)		25.1(5.5)		19.5(4.7)		17.5(3.7)	
6 per week (d)	60.9(11.0)		23.9(5.6)		18.7(4.5)		18.2(3.2)	
Number of LTCF staff		-3.36**		-1.55		-2.54*		-3.01**
<31	56.7(7.6)		23.0(3.9)		17.2(3.2)		16.5(2.9)	
≥31	62.9(10.3)		24.8(5.7)		19.7(4.7)		18.5(3.3)	

Religion, mini-mental status examination, geriatric depression scale, room type, natural window view, length of LTCF stay and frequency of family visit were not significantly associated with sense of coherence and any core component by using one-way ANOVA, independent t test or Pearson’s correlation analysis.

SD: standard deviation; LTCF: long-term care facility.

^§^*F/t/r*: One-way ANOVA(with Tukey’s post-hoc test, if needed), independent t test, or Pearson’s correlation analysis was used as appropriate.

*P<0.05

**P<0.01

Table 3 lists the results of multiple regression analysis. In Model 1 (F = 4.495, p<0.001), education level and ADL were independently associated with SOC, and these associations remained unchanged in Model 2. After controlling for personal factors, we observe a significant association between SOC and the number of LTCF staff in Model 2 (F = 4.355, p<0.001). The explanatory power for SOC increased from Model 1 (adjusted R^2^ = 0.213) to Model 2 (adjusted R^2^ = 0.264). In Model 3 (F = 12.584, p<0.001), the result of stepwise multiple regression analysis revealed that ADL and two interaction variables (high school and above education level X outdoor public space; age X number of LTCF staff) accounted for 25.2% of the variance in SOC. Model 3 had the lowest AIC value (425.755) among all models. Multicollinearity diagnostics were examined and variance inflation factors between any variables were less than 7.1 in all models.

**Table 3:** Multiple regression analysis of sense of coherence scale among participants (n = 104).

	Model 1^a^		Model 2^a^		Model 3^b^	
	B (Beta)	p value	B (Beta)	p value	B (Beta)	p value
**Main effects**						
*Personal Factor*						
Age (year)	0.164 (0.129)	0.189	0.157 (0.124)	0.203		
Marital status						
Unmarried (ref.)						
Married	-2.065 (-0.118)	0.475	-1.634 (-0.093)	0.562		
Divorce/Widowed	0.846 (0.047)	0.781	-0.425 (-0.024)	0.887		
Education level						
Illiterate (ref.)						
Elementary school	3.367 (0.184)	0.056	2.969 (0.163)	0.100		
High school and above	7.191 (0.289)	**0.004**	5.434 (0.218)	**0.028**		
Multi-morbidity	-0.155 (-0.028)	0.759	-0.181 (-0.033)	0.716		
Activities of daily living	0.148 (0.266)	**0.004**	0.169 (0.304)	**0.003**	0.189 (0.339)	**<0.001**
Voluntary admission (yes [ref.] /no)	3.072 (0.148)	0.098	2.883 (0.139)	0.110		
*Physical Environmental Factor*						
Outdoor public space (no [ref.]/yes)			-0.829 (-0.045)	0.843		
*Social Environmental Factor*						
Number of leisure activities per week			-1.247 (-0.142)	0.525		
Number of LTCF staff (<31 [ref.]/ ≥31)			8.443 (0.428)	**0.017**		
**Interaction effects**						
Education level (High school and above) x Outdoor public space					3.028 (0.180)	**0.040**
Age x Number of LTCF staff					0.002 (0.361)	**<0.001**
Adjusted R^2^	0.213		0.264		0.252	
Akaike information criterion	431.672		427.459		425.755	

LTCF: long-term care facility. p value less than 0.05 in bold.

^a^ Hierarchical regression analysis with personal and environmental factors entered as covariates block-wise into the models.

^b^ Stepwise regression analysis using personal and environmental factors and their interactions.

## Discussion

In the present study, we examined SOC status and its three core components among older adult LTCF residents in northeastern Taiwan and investigated how SOC is influenced by personal and environmental factors. Education level, ADL score and number of LTCF staff were demonstrated to be the main determinants of SOC. In addition, significant interactive effects between personal and environmental factors on SOC could be observed. Knowledge of SOC status among older adults in Taiwan is limited, especially for those residing in LTCFs. Our study may contribute to the understanding of SOC status among this specific older adult population.

The mean score (±SD) of the 13-item Chinese SOC scale among our participants was 58.3 (±8.8), which is similar to results (mean score 57.7 ±8.0) of a study performed on Hong Kong Chinese adults with diabetes [27] and is also very close to the normative data reported by Antonovsky [9]. Antonovsky cited means on normative data from published literatures using SOC-13 scale ranging from mean values 58.5 to 68.7 [9]. However, our results were lower than the nursing home residents’ mean SOC-13 scores reported in Western countries. Cole found a mean score of 65.5 among nursing home residents aged 72–88 years in the United States [28]. A study in Norway that included older people (mean age 85.4 years) staying in nursing homes also showed the mean SOC-13 scores as high as 69.1 [29]. Antonovsky stated that an individual’s SOC is constructed within his or her own cultural, social and historical background [10]. The SOC variation across studies could be due to the influence of differences in subjective experiences or cultural backgrounds on an individual’s intrinsic beliefs. For example, SOC is positively associated with optimism [19]. However, there is cultural variation on optimistic and pessimistic bias [30]. Westerners held an optimistic bias in the prediction of positive and negative events, whereas Easterners held a pessimistic bias for negative events. Therefore, we suggest conducting further study to explore the cultural influence on SOC between Eastern and Western older adults.

The present study revealed that environmental resources can still influence SOC status after personal factors are controlled for. The number of LTCF staff was independently associated with older adult residents’ SOC. Caring personnel can serve as an important resource for LTCF residents. Compared with family members, caring personnel have more consistent and prolonged contact with residents and provide overall care services including nursing, nutrition, rehabilitation, and social work. Because of the mandatory requirement for the nursing staffing ratio in Taiwan [24], the number of staff is relatively proportional to the accommodation capacity of LTCFs. The impact of LTCF size or staff number on residents’ SOC has not been studied in detail. Similarly, the association between facility size and nursing home residents’ quality of life is still inconsistent in the literature [31]. We speculate that larger sized LTCFs with more skilled staff members may be more likely to offer an engaging social environment characterized by comprehensive care, better orientation, increased social interaction and support. In the present study, even with similar staffing ratio, facilities with a staff number ≥31 held more leisure activities per week than those with a staff number <31 (5.6 ±0.5 vs. 3.8 ±0.6, p< 0.001). In addition, with more staff members available to help them meet the demands imposed by contextual stimuli, LTCF residents may demonstrate a higher degree of manageability. Indeed, we discovered that the residents of LTCFs with more staff had a significantly higher mean manageability subscale score than those who stayed in LTCFs with fewer staff (t = -2.54, p = 0.015). Furthermore, there was a trend that the former also had higher mean subscale scores of comprehensibility and meaningfulness than the latter (t = -1.55, p = 0.129; t = -3.01, p = 0.003, respectively). This suggests that number of staff could represent a crucial environmental factor in the process of delivering comprehensible, manageable and meaningful care service in the facilities.

Regarding personal factors, we discovered a significant trend of LTCF residents with a higher education level having higher scores in SOC and its three core components. Our results are similar to those of previous studies on older adults [32,33]. It has been clearly recognized that education is an important resource for older adults [34,35]. Knowledgeable and highly educated persons may have access to and easily obtain other sources of well-being [32].

Compared with those living at home, older adult residents of LTCFs or nursing homes are more likely to be physically or functionally disabled and in need of nursing care services. In the present study, 60.6% of participants had ADL scores ≤ 60, and our results indicated that ADL score is significantly associated with SOC across all models. Tsuno & Yamazaki demonstrated that limitations to physical activity showed significant association with SOC among both urban and rural residents in Japan after adjusting for sociodemographic characteristics [36]. Borglin *et al*. found that Swedish elderly with low present quality of life were less physically active and had lower SOC scores as well [37]. Those with greater ADL performance limitations may have less confidence in coping, resulting in a negative impact on manageability. Hence, it is worth exploring the impact of physical rehabilitation, the intervention that might improve ADL, on SOC status in future research.

Some gerontological studies have focused on the relationship between the individual and the environment, providing insight into personal and environmental factors that influence health outcomes in long-term care settings [38–40]. According to Lawton’s model of person-environmental fit [23], subjects with lower personal competence are best matched with environments with fewer demands and more support, such as a skilled nursing environment [39]. However, the impact of person-environment interaction on SOC status among older adult LTCF residents is not well understood. Our results demonstrate a statistically significant interaction effect on SOC between age and number of staff (standardized interaction coefficient = 0.361, p<0.001). This effect indicates that older adults in LTCFs with more staff may have a stronger SOC than those who stay in LTCFs with fewer staff. This is reasonable, given that people may require more caring resources while aging. In addition, we observed that environmental factors (outdoor public space) may moderate the relationship between education level and SOC (standardized interaction coefficient = 0.361, p<0.001). This suggests that more educated residents who stay in LTCFs with outdoor public space may have a stronger SOC than those who stay in LTCFs without outdoor public space. Although the mechanism of these interaction effects is unclear and must be studied further, it seems that the effects of personal factors on SOC status are not constant but vary depending upon the environmental context. The explanatory power of Model 2 (the full model) was slightly higher than that of Model 3 (with interaction terms included). However, Model 3 had the lowest AIC value among the three regression models. The lower the corresponding AIC value is, the better the fit of the model is, and the less information loss the model has [41]. The present pilot study illustrates the importance of utility of multidimensional perspective and integration of the influence of personal-environmental interaction for SOC evaluation.

Several limitations in the present study should be addressed. First, because of the inherent disadvantage of cross-sectional study design, it is impossible to infer a causal relationship between SOC status, personal and environmental factors. We can only interpret the results as associations. Second, though we surveyed 46.2% officially certified LTCFs in Yilan County, sampling was done with a nonprobability method, creating a potential shortcoming in terms of representativeness. Finally, given that all of the participants were of Chinese ethnicity, further evaluation is necessary for the implications of our results to be generalized in other contexts.

### Conclusion

The present study provides evidence regarding the SOC status and association factors among older adult LTCF residents in Yilan, Taiwan. To our knowledge, this is the first study reporting the SOC of institutionalized older adults in Taiwan. Our findings indicate that older adult LTCF residents in Taiwan had relatively low SOC scores compared to their counterparts in Western countries. Among personal and environmental factors, we discovered that education level, ADL score and number of LTCF staff were factors significantly associated with the SOC status. Those who had a higher education level and higher ADL scores and those who stayed in LTCFs with more staff had a stronger SOC. In addition, our findings suggest that comprehensive elevation of SOC among older adult LTCF residents should integrate the influence of interactions between personal and environmental factors. The challenge for LTCF staff is to help residents maintain or strengthen their SOC. Although this pilot study has some limitations, our results may be helpful for LTCF staff to gain insight into the impact of environmental factors on SOC and prompt them to pay more attention to personal-environmental interaction effects. More longitudinal studies with a larger sample size are warranted to achieve a greater understanding of the relationship between SOC, personal characteristics and environmental resources of older adults in long-term care settings.

## Supporting Information

S1 Table Univariable regression analysis of sense of coherence scale and three core components among participants (n = 104). (DOC)
