# Supplementary material for: Sense of Coherence among Older Adult Residents of Long-Term Care Facilities in Taiwan: A Cross-Sectional Analysis
Source: PLoS One. 2016 Jan 11;11(1):e0146912. doi: 10.1371/journal.pone.0146912 (PMC4709091; doi:10.1371/journal.pone.0146912)
Supplement: S1 Table — (DOC) [file pone.0146912.s001.doc]

S1 Table. Univariable regression analysis of sense of coherence scale and three core components among participants (n=104).

|  | SOC | | Comprehensibility | | Manageability | | Meaningfulness | |
| --- | --- | --- | --- | --- | --- | --- | --- | --- |
|  | β | p value | β | p value | β | p value | β | p value |
| **Personal Factor** |  |  |  |  |  |  |  |  |
| Age (year) | 0.263 | **0.028** | 0.133 | **0.029** | 0.103 | **0.047** | 0.028 | 0.516 |
| Gender (female [ref.]/male) | 0.022 | 0.990 | 0.150 | 0.869 | -1.331 | 0.082 | 1.228 | 0.053 |
| Marital status |  |  |  |  |  |  |  |  |
| Unmarried (ref.) |  |  |  |  |  |  |  |  |
| Married | -2.519 | 0.390 | -0.859 | 0.563 | -0.144 | 0.910 | -1.496 | 0.171 |
| Divorce/Widowed | 2.975 | 0.323 | 2.025 | 0.186 | 2.000 | 0.128 | -1.050 | 0.348 |
| Education level |  |  |  |  |  |  |  |  |
| Illiterate (ref.) |  |  |  |  |  |  |  |  |
| Elementary school | 3.143 | 0.085 | 1.596 | 0.092 | 0.982 | 0.230 | 0.546 | 0.396 |
| High school and above | 8.314 | **0.001** | 3.196 | **0.014** | 1.778 | 0.111 | 3.321 | **<0.001** |
| Religion (no [ref.]/yes) | 1.710 | 0.501 | 1.160 | 0.369 | 0.868 | 0.429 | -0.308 | 0.736 |
| Multi-morbidity | 0.371 | 0.497 | 0.126 | 0.651 | -0.029 | 0.903 | 0.270 | 0.167 |
| Activities of daily living | 0.174 | **0.001** | 0.073 | **0.008** | 0.084 | **<0.001** | 0.017 | 0.378 |
| Mini-mental status examination | -0.052 | 0.929 | -0.043 | 0.885 | -0.332 | 0.189 | 0.316 | 0.131 |
| Geriatric depression scale | 0.335 | 0.507 | 0.209 | 0.417 | -0.001 | 0.997 | 0.124 | 0.495 |
| Voluntary admission (yes [ref.] /no) | 3.417 | 0.095 | 2.296 | **0.027** | -0.029 | 0.974 | 1.163 | 0.114 |
| **Physical Environmental Factor** |  |  |  |  |  |  |  |  |
| Room type |  |  |  |  |  |  |  |  |
| Single room (ref.) |  |  |  |  |  |  |  |  |
| Double room | -1.321 | 0.679 | -1.476 | 0.361 | 0.214 | 0.878 | -0.060 | 0.959 |
| ≥3 persons/room | -3.144 | 0.255 | -2.347 | 0.094 | 0.026 | 0.983 | -0.809 | 0.415 |
| Natural window views (no [ref.]/yes) | 0.070 | 0.972 | -0.385 | 0.699 | 1.028 | 0.222 | -0.560 | 0.424 |
| Outdoor public space (no [ref.]/yes) | 4.747 | **0.009** | 1.630 | 0.079 | 1.839 | **0.019** | 1.306 | **0.045** |
| **Social Environmental Factor** |  |  |  |  |  |  |  |  |
| Length of LTCF stay (months) | 0.047 | 0.348 | 0.036 | 0.151 | 0.023 | 0.292 | -0.011 | 0.520 |
| Number of leisure Activities per week | 1.015 | 0.242 | 0.068 | 0.878 | 0.505 | 0.178 | 0.449 | 0.148 |
| Number of family visits per week | 0.712 | 0.653 | 0.956 | 0.234 | 0.663 | 0.332 | -0.886 | 0.117 |
| Number of LTCF staff (<31 [ref.]/ ≥31) | 6.235 | **0.001** | 1.803 | 0.068 | 2.442 | **0.003** | 2.026 | **0.003** |

Abbreviations: SOC: sense of coherence. LTCF: long-term care facility.

p value less than 0.05 in bold.
